# Supplementary material for: Network Pharmacology of Ginseng (Part III): Antitumor Potential of a Fixed Combination of Red Ginseng and Red Sage as Determined by Transcriptomics
Source: Pharmaceuticals (Basel). 2022 Oct 30;15(11):1345. doi: 10.3390/ph15111345 (PMC9696821; doi:10.3390/ph15111345)
Supplement: Supplementary file 1 [file pharmaceuticals-15-01345-s001.zip › figure S2.pdf]

Expression Analysis - SAL

Summary | Graphical Summary | Canonical Pathways | Upstream Analysis | Diseases & Functions | Regulator Effects | Networks | Lists | My Pathways | Molecules | Analysis Match

Chart | Overlapping

Customize Chart | Horizontal Bar Chart

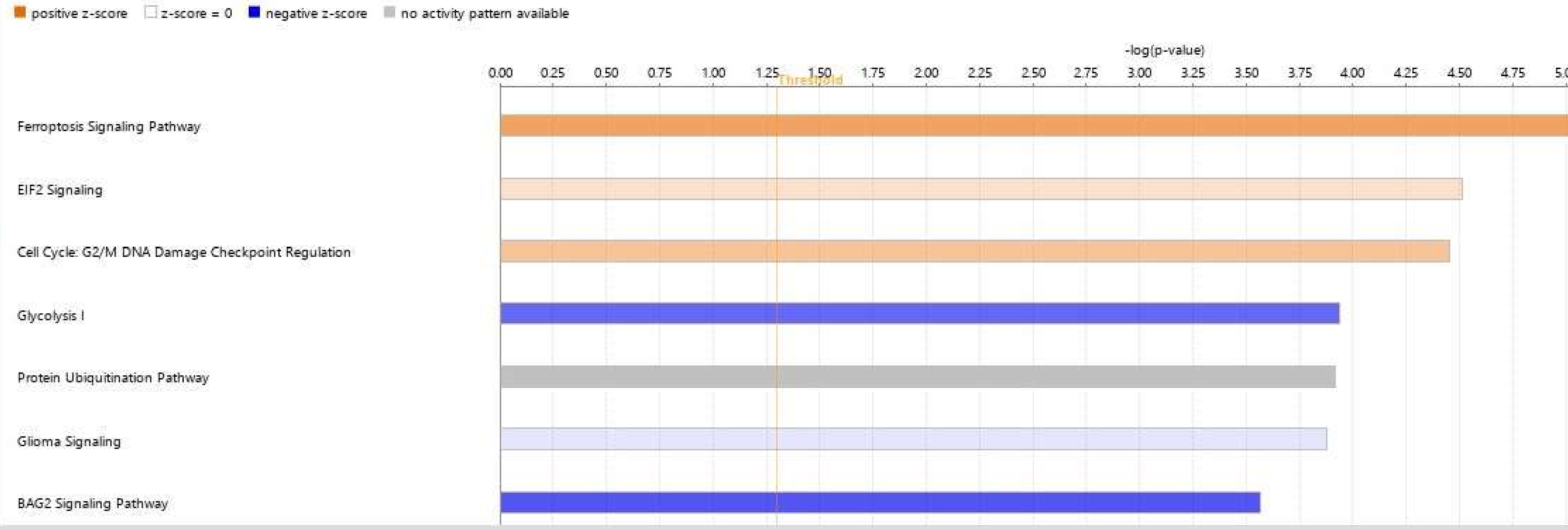

7 molecule(s) associated with **Glycolysis I** [Ratio: 7/21 (0.333)] [z-score: -1.89] [p-value: 1.15E-04]

Activity Plot | View Repo

Add To My Pathway | Add To My List | Create Dataset | Customize Table | Expand

Symbol	Entrez Gene Name	Identifier<br>Gene Symbol - hu...	Measurement<br>Expr Fold Change	Expected	Location	Type(s)	Biomarker Applica...
ALDOA	aldolase, fructose-bisphosphate A	ALDOA	↓-855.146	↑ Up	Cytoplasm	enzyme	unspecified applic... ...all
BPGM	bisphosphoglycerate mutase	BPGM	↓-37.688	↑ Up	Extracellular Space	phosphatase	
ENO1*	enolase 1	ENO1*	↓-2561.827	↑ Up	Cytoplasm	enzyme	diagnosis ...all
GPI*	glucose-6-phosphate isomerase	GPI*	↑371.745	↑ Up	Extracellular Space	enzyme	unspecified applic... ...all
PGK1*	phosphoglycerate kinase 1	PGK1*	↓-1425.541	↑ Up	Cytoplasm	kinase	
PKM	pyruvate kinase M1/2	PKM	↓-1094.053	↑ Up	Cytoplasm	kinase	unspecified applic... ...all
TPI1*	triosephosphate isomerase 1	TPI1*	↓-810.284	↑ Up	Cytoplasm	enzyme	diagnosis, unspeci... ...all
