# Supplementary material for: Network Pharmacology of Ginseng (Part III): Antitumor Potential of a Fixed Combination of Red Ginseng and Red Sage as Determined by Transcriptomics
Source: Pharmaceuticals (Basel). 2022 Oct 30;15(11):1345. doi: 10.3390/ph15111345 (PMC9696821; doi:10.3390/ph15111345)
Supplement: Supplementary file 1 [file pharmaceuticals-15-01345-s001.zip › figure S3.pdf]

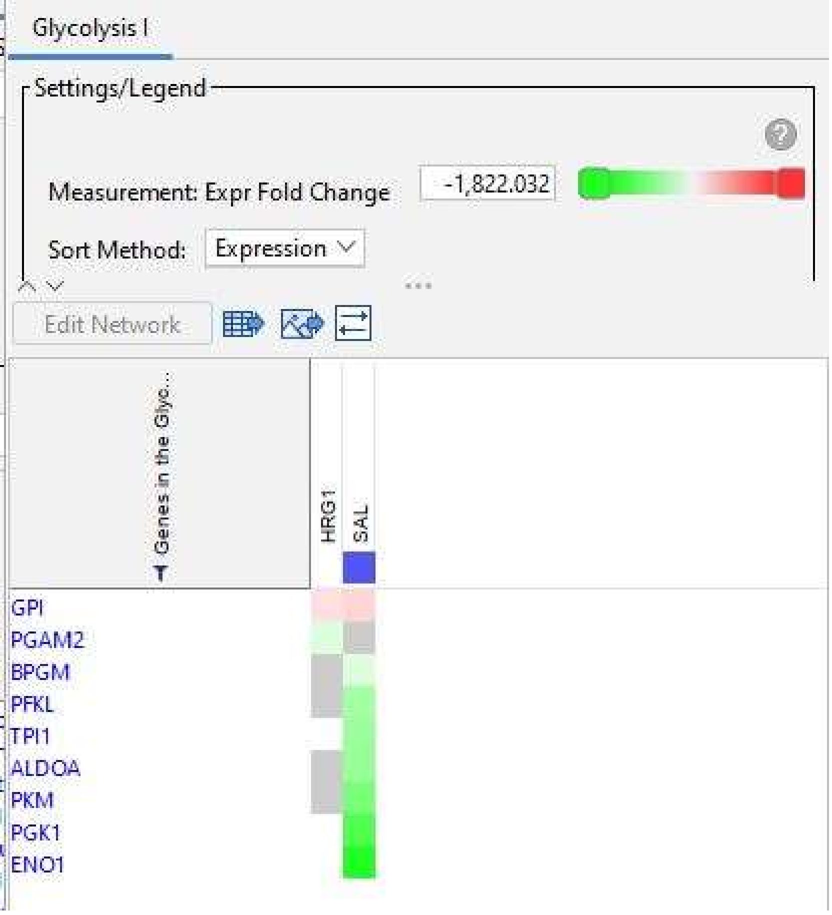
Glycolysis I
Settings/Legend
Measurement: Expr Fold Change
-1,822.032
Sort Method:
Expression
Edit Network
Genes in the Glyc...
HRG1
SAL
GPI
PGAM2
BPGM
PFKL
TPI1
ALDOA
PKM
PGK1
ENO1
