# Supplementary figures and images for: Network Pharmacology of Ginseng (Part III): Antitumor Potential of a Fixed Combination of Red Ginseng and Red Sage as Determined by Transcriptomics
Source: Pharmaceuticals (Basel). 2022 Oct 30;15(11):1345. doi: 10.3390/ph15111345 (PMC9696821; doi:10.3390/ph15111345)

positive z-score    z-score = 0    negative z-score    no activity pattern available

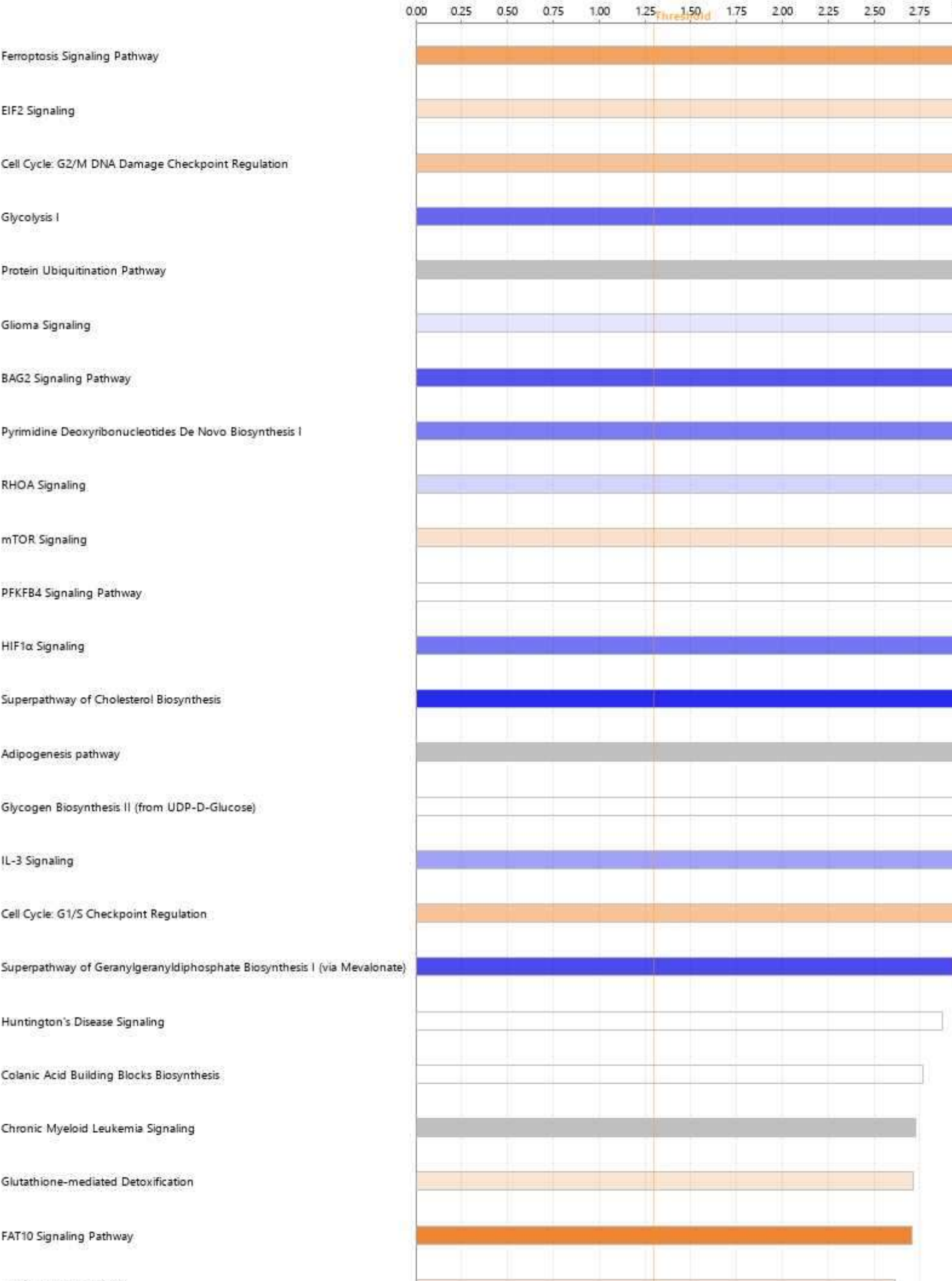

Supplement: Supplementary file 1 [file pharmaceuticals-15-01345-s001.zip › figure S1.pdf]
